# Machine learning based models for predicting presentation delay risk among gastric cancer patients

**DOI:** 10.3389/fonc.2024.1503047

**Published:** 2025-01-13

**Authors:** Huali Zhou, Qiong Gu, Rong Bao, Liping Qiu, Yuhan Zhang, Fang Wang, Wenlian Liu, Lingling Wu, Li Li, Yihua Ren, Lei Qiu, Qian Wang, Gaomin Zhang, Xiaoqing Qiao, Wenjie Yuan, Juan Ren, Min Luo, Rong Huang, Qing Yang

**Affiliations:** ^1^ School of Nursing, Chengdu Medical College, Chengdu, China; ^2^ Department of Gastric Surgery, Sichuan Clinical Research Center for Cancer, Sichuan Cancer Hospital & Institute, Sichuan Cancer Center, Affiliated Cancer Hospital of University of Electronic Science and Technology of China, Chengdu, China; ^3^ Department of General Surgery, Fourth People’s Hospital of Zigong City, Zigong, China; ^4^ Gastroenterology, Chengdu Seventh People’s Hospital, Chengdu, China; ^5^ Department of General Surgery, Meishan Hospital of Traditional Chinese Medicine, Affiliated Meishan Hospital of Chengdu University of Traditional Chinese Medicine, Meishan, China; ^6^ School of Nursing, Chuanbei Medical College, Nanchong, China; ^7^ Nursing Department, Sichuan Clinical Research Center for Cancer, Sichuan Cancer Hospital & Institute, Sichuan Cancer Center, Affiliated Cancer Hospital of University of Electronic Science and Technology of China, Chengdu, China

**Keywords:** gastric cancer, presentation delay, risk prediction, machine learning, prediction model

## Abstract

**Objective:**

Presentation delay of cancer patients prevents the patient from timely diagnosis and treatment leading to poor prognosis. Predicting the risk of presentation delay is crucial to improve the treatment outcomes. This study aimed to develop and validate prediction models of presentation delay risk in gastric cancer patients by using various machine learning models.

**Methods:**

875 cases of gastric cancer patients admitted to a tertiary oncology hospital from July 2023 to June 2024 were used as derivation cohort, 200 cases of gastric cancer patients admitted to other 4 tertiary hospital were used as external validation cohort. After collecting the data, statistical analysis was performed to identify discriminative variables for the prediction of presentation delay and 13 statistically significant variables are selected to develop machine learning models. The derivation cohort was randomly assigned to the training and internal validation set by the ratio of 7:3. Prediction models were developed based on six machine learning algorithms, which are logistic regression (LR), support vector machine (SVM), random forest (RF), gradient boosted trees (GBDT), extremely gradient boosting (XGBoost) and muti-layer perceptron (MLP). The discrimination and calibration of each model were assessed based on various metrics including accuracy, sensitivity, specificity, positive predictive value (PPV), negative predictive value (NPV), F1-Score and area under curve (AUC), calibration curves and Brier scores. The best model was selected based on comparing of various metrics. Based on the selected best model, the impact of features to the prediction result was analyzed with the permutation feature importance method.

**Results:**

The incidence of presentation delay for gastric cancer patients was 39.3%. The developed models achieved performance metrics as AUC (0.893-0.925), accuracy (0.817-0.847), sensitivity (0.857-0.905), specificity (0.783-0.854), PPV (0.728-0.798), NPV (0.897-0.927), F1 score (0.791-0.826) and Brier score (0.107-0.138) in internal validation set, which indicated good discrimination and calibration for the prediction of presentation delay in gastric cancer patients. Among all models, RF based model was selected as the best one as it achieved good discrimination and calibration performance on both of internal and external validation set. Feature ranking results indicated that both of subjective and objective factors have significant impact on the occurrence of presentation delay in gastric cancer patients.

**Conclusion:**

This study demonstrated that the RF based model has favorable performance for the prediction of presentation delay in gastric cancer patients. It can help medical staffs to screen out high-risk gastric cancer patients for presentation delay, and to take appropriate and specific interventions to reduce the risk of presentation delay.

## 1 Introduction

Gastric cancer is one of the common malignant tumors of the digestive system, and the incidence and mortality rates rank the 5th and 3rd of malignant tumors worldwide, respectively (1). As of 2022, the incidence rate of gastric cancer in China reaches 35.87/100,000, ranking the 4th and 6th among malignant tumors in men and women respectively. The mortality rate of gastric cancer is 26.04/100,000, ranking the 3rd and 4th among malignant tumors in men and women respectively (2). The five-year survival rate of early gastric cancer can reach 90% and above. However, about 90% of the gastric cancer patients in China are in the progressive stage at the time of diagnosis, and the five-year survival rate is less than 30% (3). Previous studies had shown that the prognosis of gastric cancer is closely related to the timing of diagnosis and treatment (4).

The concept of medical delay was first proposed by Pack and Gallo in 1938 and can be divided into two kinds, i.e. symptom to presentation delay (SPD) and presentation to treatment delay (PTD) (5). Among them, presentation delay refers to the time between the first detection of suspicious symptoms related to cancer and the patient’s first visit to a healthcare facility is more than 3 months (5). Some studies have shown that patients are generally treated effectively during the diagnosis period after presentation (6). Therefore, treatment delay has less impact on patients than presentation delay. Domestic and international studies have shown that the incidence of presentation delay for cancer patients ranges from 33% to 54% (6, 7). In rural counties of four provinces in China (Henan, Shandong, Jiangsu, and Anhui), the average time of delay in presentation for cancers of the upper gastrointestinal tract (stomach and esophagus) is 119 days, and the incidence of presentation delay (≥3 months) was 30.0% (8, 9). Presentation delay not only leads to poor prognosis of patients with gastric cancer, affects the patient’s quality of life and survival time, but also increases medical costs. Therefore, it is important to prevent presentation delay and improve the prognosis of gastric cancer patients.

Risk prediction is one effective way to prevent patient presentation delay. Based on the prediction results, populations with high presentation delay risk can be identified timely (10). On one hand, it helps patients to improve the perception of risk on delayed access to healthcare. On the other hand, it enhances the ability of medical staff to identify high-risk population in an early stage and optimize resource allocation (11, 12). With the rapid development of artificial intelligence (AI) techniques, powerful machine learning (ML) models have been introduced into medical research and help to develop medical prediction models with improved performance (13). By using various diseases related factors such as demographic and pathologic data, ML models were used to predict the probability of certain patient outcomes, such as incidence and recurrence of diseases (11, 12, 14, 15).

Previous researches on presentation delay of gastric cancer patients were mostly limited to current situation analysis and influence factors investigation (4, 16–18). Although there are some studies on prediction model for diagnosis/treatment delay of cancer patients (19, 20), to the best of our knowledge there is no prediction model for presentation of gastric cancer patients. The objective of this study was to explore and validate various ML methods for constructing predictive models for presentation delay of gastric cancer patients. Self-developed questionnaires and authoritative scales were used to collect gastric cancer patient information that were supposed to be highly correlated with the occurrence of presentation delay. The collected data included demographic information, health-related information, medical treatment history, family support level, health literacy management knowledge, medical coping modes and emotional states. Based on the clinical data, the influence factors of presentation delay in gastric cancer patients were firstly analyzed, revealing 13 statistically significant variables: ethnicity, age, education, place of residence, medical insurance, regular medical examination, family support score, health literacy score, medical coping modes (confrontation, avoidance, resignation), anxiety scores and depression scores. Based on those selected variables, six predictive models were constructed and validated. It was hoped that the constructed models can provide useful tools for nurses to screen the high-risk groups and reduce the risk of presentation delay of gastric cancer patients.

## 2 Materials and methods

### 2.1 Patients

From July 2023 to June 2024, 875 cases of gastric cancer patients admitted to a tertiary oncology hospital (Sichuan Cancer Hospital & Research Institute) in Sichuan Province, China were selected for cross-sectional study. The inclusion criteria were: (1) clear consciousness, normal language expression ability and comprehension; (2) age ≥ 18 years; (3) voluntary participation in this study; (4) gastric cancer patients diagnosed by pathology. The exclusion criteria were: (1) people with comprehension or reading disabilities; (2) patients with other cancers in combination; (3) patients with non-primary gastric cancer. 200 gastric cancer patients admitted to other four tertiary hospitals in Sichuan Province from July 2023 to June 2024 were selected as external validation data. The study was reviewed and approved by the Medical Ethics Committee of the hospital (Approval No. SCCHEC-02-2023-127) after informed consent was obtained and signed by the patients.

### 2.2 Data collection procedure

We conducted a survey-based data collection procedure. The survey team consisted of more than ten clinical nurses who had worked for more than 10 years and had undergone unified training before collecting data. They used self-developed questionnaires and authoritative scales to collect data on gastric cancer patients by distributing questionnaires or one-on-one consultation. The used questionnaires/scales include:

1. Demographic information questionnaire: A self-developed questionnaire on general demographic information, including gender, ethnicity, age, education, type of household, occupation, total household income, marital status, and form of payment for medical care.
2. Health related questionnaire: A self-developed health-related questionnaires containing information on alcohol consuming, preference of stimulating/smoky/fried/pickled foods, family history of stomach cancer, physical examination situation, and chronic gastric disease status.
3. Medical treatment questionnaire: A self-developed questionnaire that includes: choice of hospital for the first visit, clinical stage and pathological type of gastric cancer, the initial symptom of gastric discomfort and the first time of detection, the first time of seeking medical treatment. The delay of seeking medical treatment was assessed by the investigator based on whether the time between the patient’s first symptom and visit to the healthcare facility was ≥90 d.
4. Family support scale (FSS): The family support scale was improved by Wang Guorong et al. (21) based on the scale developed by Procidana and Heller (22). This scale contains 15 entries, forming a three-level scale, including “fully compliant = 3 points”, “partially compliant = 2 points”, and “not at all compliant = 1 point”. The total score ranges from 15 to 45 points, and higher scores indicating higher levels of family support. The Cronbach’s α coefficient of the questionnaire entries was measured to be 0.83.
5. Health Literacy Management Scales (HeLMS): This scale was developed by Jordan et al. (23) and modified by Haolin Sun (24). The scale consists of 24 items of 4 categories, which are information acquisition ability (9 items), communication and interaction ability (9 items), willingness to improve health (4 items), and willingness to pay (2 items). Each item has 5 level options and the total score ranges from 24 to 120. The Cronbach’s α coefficient was measured as 0.85, indicating good reliability and validity.
6. The Medical Coping Modes Questionnaire (MCMQ): This questionnaire was developed by Feifel et al. (25) and adapted by Shen et al. (26). The scale is comprised of total 24 items with four-point (1–4) Likert scales, and is divided into 3 subscales: confrontation scale (8 items), avoidance scale (7 items), and resignation scale (5 items). Higher scores of each subscale indicate that the patient tends to adopt that coping style.
7. Generalized Anxiety Disorder Scale (GAD-7): The scale is based on the seven diagnostic criteria for anxiety disorders of the Diagnostic and Statistical Manual of Mental Disorders (DSM) developed by the American Psychiatric Association (APA) (27). It has been proven to be a psychometric scale for screening, identifying, and evaluating anxiety states with good reliability, sensitivity, and specificity. The questionnaire consists of 7 questions, each of which has 4 answers that corresponds to a score of 0/1/2/3. The total score is 21, with higher scores indicating higher levels of anxiety. The Cronbach’s α coefficient was measured as 0.907.
8. Patient Health Questionnaire-9 (PHQ-9): This questionnaire is based on nine entries of the DSM-IV (Diagnostic and Statistical Manual of Mental Disorders developed by the American Psychiatric Association) diagnostic criteria (28). It is a simple and valid self-assessment scale for depressive disorders. Each question has 4 answers of score 0/1/2/3. The scale has a total score of 27, with higher scores indicating a higher degree of depression. The Cronbach’s a coefficient is 0.767, indicating good reliability and validity.

Other data such as information from medical records were combined to ensure the data were collected in a complete and reliable manner. After the questionnaires were completed, the researcher examined them one by one and asked the patient to fill up missing items if there was any. Invalid questionnaires with inconsistent and regular answers were excluded to ensure the authenticity and accuracy of the research data. During the survey, the questionnaires were registered and numbered so no missing or duplication occurred. After the survey, the completeness and correctness of the questionnaires was checked again, and timely remedial actions were taken if problems were found.

### 2.3 Statistical analysis

SPSS 26.0 and Python 3.7.1 were used to perform statistical analysis. The measurement data conforming to normal distribution were expressed in the form of $\bar{x}\;\pm \;s$ , and Student’s *t* test was used for comparison between sets. Those not conforming to normal distribution were expressed in the form of median and quartiles $M\left(P_{25},P_{75}\right)$ , and Mann−Whitney test was used for comparison. Categorical data were expressed in the form of frequency and percentage, and comparison between sets were performed with chi-square test. Variables with statistically significant differences were screened according to the criterion of P<0.05 and included in the predictive model modeling analysis.

### 2.4 Machine learning models

**Figure 1:**
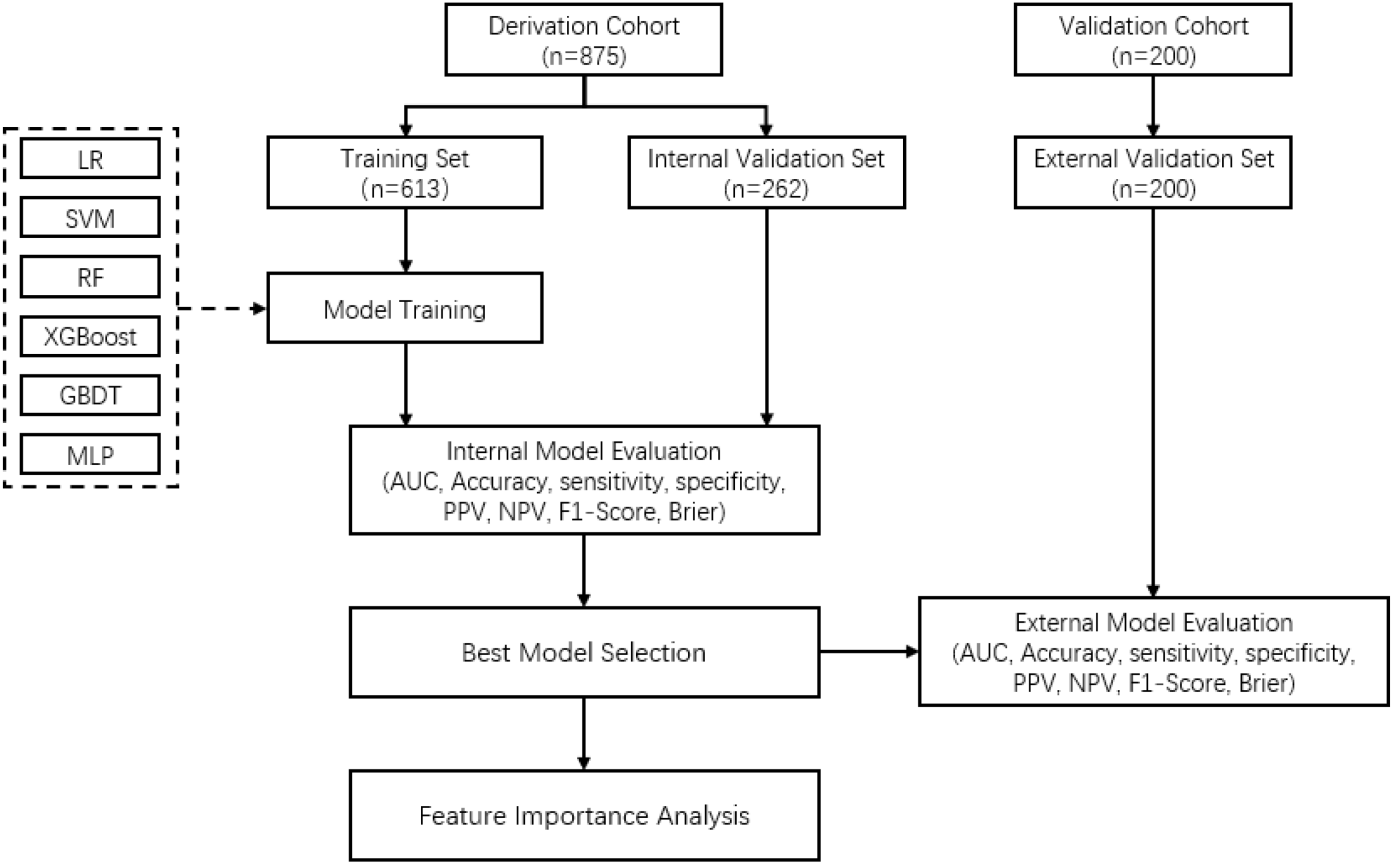
Flowchart of the model developing and validation process for prediction of presentation delay risk in gastric cancer patients.

The whole population was randomly assigned to training set and validation set according to a 7:3 ratio, resulting in a training set with 613 samples and a validation set with 262 samples. Prediction models were developed by using following machine learning methods: logistic regression (LR) (29), support vector machine (SVM) (30), random forest (RF) (31), extreme gradient boosting (XGBoost) (32), gradient boosting decision tree (GBDT) (33) and multilayer perceptron (MLP) (34). All machine learning models were developed using Python 3.7.1. For model training, 5-fold cross validation and grid search were used for optimal hyper-parameter determination. The developed models were validated and compared with the internal and external validation set by using following metrics: the area under curve (AUC) derived from receiver operating characteristic (ROC) curve, accuracy, sensitivity, specificity, positive predictive value (PPV), negative predictive value (NPV), F1-Score and Brier score. When the performance metrics were not consistent, AUC was used as the main reference metric. The models were compared and the optimal model was selected. Based on the selected optimal model, the contribution of features for the prediction of the presentation delay risk was analyzed by feature ranking. The whole process of model development and validation is shown in **Figure 1**.

## 3 Results

### 3.1 Basic characteristics of patients

According to the time between the initial perception of symptoms and the first visiting of patient to hospital, the 875 gastric cancer patients were classified as non-delayed group with 531 samples (60.7%) and delayed group with 344 samples (39.3%). There were 628 male cases (71.8%) and 247 female cases (28.2%). 774 cases (88.5%) were Han nationality and 101 cases (11.5%) were minority nationality. 395 cases (45.1%) were over 60 years old, and 480 cases (54.9%) were under 60 years old. Patients with high-school or above level education amounted to 195 (22.3%) and other 680 patients (77.7%) had education level below than high school. With regard to the medical insurance type, there were 244 cases (27.9%) of basic medical insurance for employees, 163 cases (18.6%) of basic medical insurance for urban residents, 444 cases (50.7%) of new rural cooperative medical insurance, and 24 cases (2.8%) of other insurance types. Patients lived in urban and rural area were 324 (37.0%) and 551 (63.0%) respective. There were 518 (59.2%) patients that enjoy spicy, smoked, fried or pickled foods, and 632 (72.2%) patients have never undergone a physical examination.

### 3.2 Included features

The data of the delayed and non-delayed groups were compared with statistical tests. The results indicated that the differences between the two groups in ethnicity, age, education, place of residence, medical insurance, regular medical examination, family support score (FSS), health literacy score (HeLMS), medical coping modes (confrontation-MCMC, avoidance-MCMA, resignation-MCMR), anxiety scores (GAD-7) and depression scores (PHQ-9) were statistically significant (P<0.05). The aforementioned 13 statistically significant features were included for ML models training and testing. For continuous variables, the raw data were used directly. For categorical variables, the data were processed by binary encoding or dummy encoding. The details of statistical analysis results and feature encoding are shown in **Table 1**.

**Table 1:** Statistical analysis results and feature encoding of the derivation cohort (n = 875).

Variable	Variable Encoding	Non-Delayed Group (n=531)	Delayed Group (n=344)	Statistic	*P*
Ethnic group				$\chi^{2}$ =7.09	0.008
Han	0	482 (90.8%)	292 (84.9%)		
Minority	1	49 (9.2%)	52 (15.1%)		
Age				$\chi^{2}$ =4.52	0.033
<60	0	276 (52.0%)	204 (59.3%)		
≥60	1	255 (48.0%)	140 (41.7%)		
Education Level				$\chi^{2}$ =4.43	0.035
Below high-school	0	400 (75.3%)	280 (81.4%)		
High-school or above	1	131 (24.7%)	64 (18.6%)		
Place of residence				$\chi^{2}$ =27.2	<0.001
Rural area	0	298 (56.1%)	253 (73.5%)		
Urban area	1	233 (43.9%)	91 (26.5%)		
Medical Insurance Type				$\chi^{2}$ =15.6	0.001
Medical insurance for employees	[1,0,0]	171 (32.2%)	73 (21.2%)		
Basic medical insurance for urban residents	[0,1,0]	102 (19.2%)	61 (17.7%)		
New rural cooperative medical insurance	[0,0,1]	246 (46.3%)	198 (57.6%)		
Other (Commercial/Medical assistance/self-funded, etc)	[0,0,0]	12 (2.3%)	12 (3.5%)		
Regular Medical Examination				$\chi^{2}$ =11.07	<0.001
No	0	362 (68.2%)	270 (78.4%)		
Yes	1	169 (31.8%)	74 (21.6%)		
Family support score $\bar{x}\pm s$	——	11.23 ± 2.19	9.77 ± 2.17	t =9.64	<0.001
Health literacy score (HeLMS) $\bar{x}\pm s$	——	91.0 ± 17.07	82.11 ± 17.61	t =7.42	<0.001
Anxiety score (GAD-7) $M\left[P_{25},P_{75}\right]$	——	3 [1, 5]	1 [0, 4]	Z=7.81	<0.001
Depression score (PHQ-9) $M\left[P_{25},P_{75}\right]$	——	2 [0, 5]	5 [2, 9]	Z=-7.31	<0.001
Medical Coping Mode-Confrontation (MCMC) $\bar{x}\pm s$	——	19.72 ± 2.26	18.38 ± 2.55	t =8.13	<0.001
Medical Coping Mode-Avoidance (MCMA) $\bar{x}\pm s$	——	16.12 ± 2.43	17.35 ± 2.47	t =7.29	<0.001
Medical Coping Mode- Resignation (MCMR) $\bar{x}\pm s$	——	12.53 ± 1.67	13.67 ± 1.81	t =9.62	<0.001

### 3.3 Model development and internal validation

Using the selected features as input, six machine learning models, i.e., LR, SVM, RF, XGBoost, GBDT and MLP, were trained and evaluated for predicting the presentation delay risk of gastric cancer patients. During development of models, 70% of the whole dataset were randomly selected for training. By combine the strategy of 5-fold cross validation and grid search, the optimal hyper-parameters for each model were determined. Then the model was retrained with the determined hyper-parameters and the whole training set to obtain the final model.

**Figure 2:**
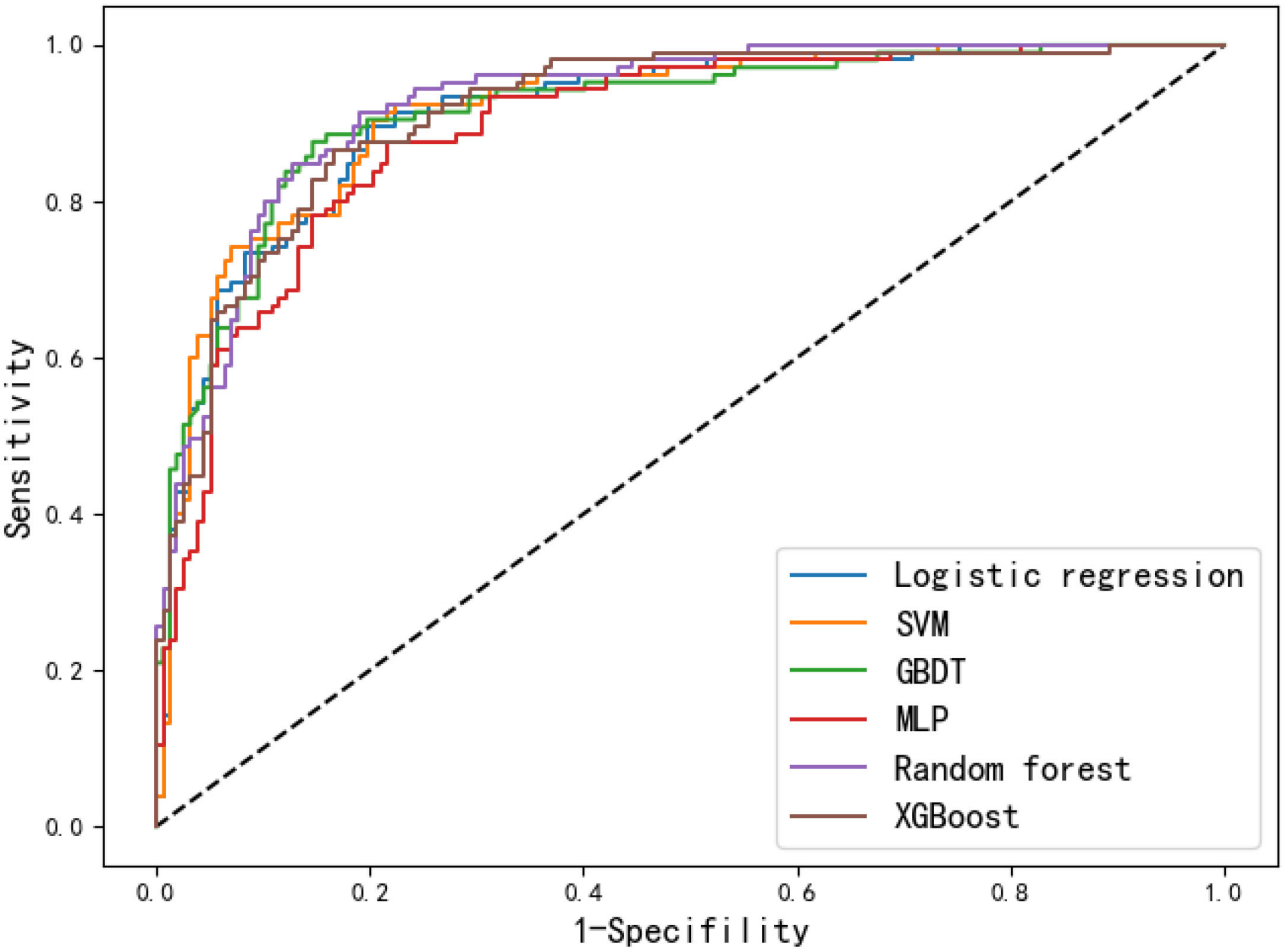
Receiver operating curves of the six models on the internal validation set.

The performance metrics of each model on training and internal validation set are summarized in **Table 2**. The receiver operating curves (ROC) are shown in **Figure 2**, based on which the AUC values were derived. By comparing the performance metrics on training and internal validation set, it can be seen that the gaps between metrics such as AUC, accuracy and F1-score are relatively small. The only exception if the MLP based model, which had relative larger performance gap than the other models. The reason might be that MLP is well known to be prone to overfitting. The risk of overfitting is low for all the other models.

**Table 2:** Evaluation metrics of different ML models on training and validation set.

Metric	Training Set						Internal Validation Set					
	LR	SVM	RF	XGBoost	GBDT	MLP	LR	SVM	RF	XGBoost	GBDT	MLP
AUC	0.889	0.892	0.934	0.923	0.929	0.927	0.910	0.915	0.925	0.915	0.914	0.893
Accuracy	0.827	0.825	0.848	0.840	0.874	0.843	0.836	0.836	0.847	0.844	0.859	0.817
Sensitivity	0.736	0.774	0.879	0.904	0.858	0.908	0.886	0.895	0.905	0.857	0.867	0.867
Specificity	0.885	0.858	0.829	0.799	0.885	0.802	0.803	0.796	0.809	0.834	0.854	0.783
PPV	0.804	0.777	0.766	0.742	0.827	0.745	0.750	0.746	0.760	0.776	0.798	0.728
NPV	0.840	0.856	0.914	0.929	0.907	0.932	0.913	0.919	0.927	0.897	0.905	0.898
F1 Score	0.769	0.776	0.819	0.815	0.842	0.819	0.812	0.814	0.826	0.814	0.831	0.791
Brier	0.126	0.124	0.099	0.106	0.099	0.101	0.117	0.114	0.107	0.115	0.111	0.138

**Figure 3:**
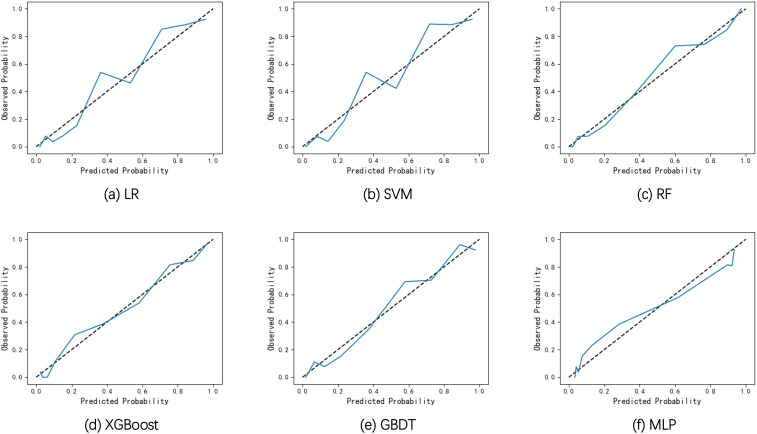
Calibration curves of the six models on the internal validation set. **(A)** Calibration curves of LR. **(B)** Calibration curves of SVM. **(C)** Calibration curves of RF. **(D)** Calibration curves of XGBoost. **(E)** Calibration curves of GBDT. **(F)** Calibration curves of MLP.

The discrimination performance of the developed models was generally well, which is indicated by the values of AUC (0.893-0.925), accuracy (0.817-0.847), sensitivity (0.857-0.905), specificity (0.783-0.854), PPV (0.728-0.798), NPV (0.897-0.927) and F1 score (0.791-0.826) on the internal validation set. To better demonstrate the calibration degree of different models, the calibration curves are also shown in **Figure 3**. The calibration curves and the Brier scores in **Table 2** indicate that the models were well calibrated. Moreover, based on the evaluation results, RF was found to have the best performance with respect to the AUC metric (AUC=0.925). The RF model also had the best sensitivity (0.905) and Brier score (0.107), and the second-best accuracy value (82.8%).

### 3.4 External validation

**Figure 4:**
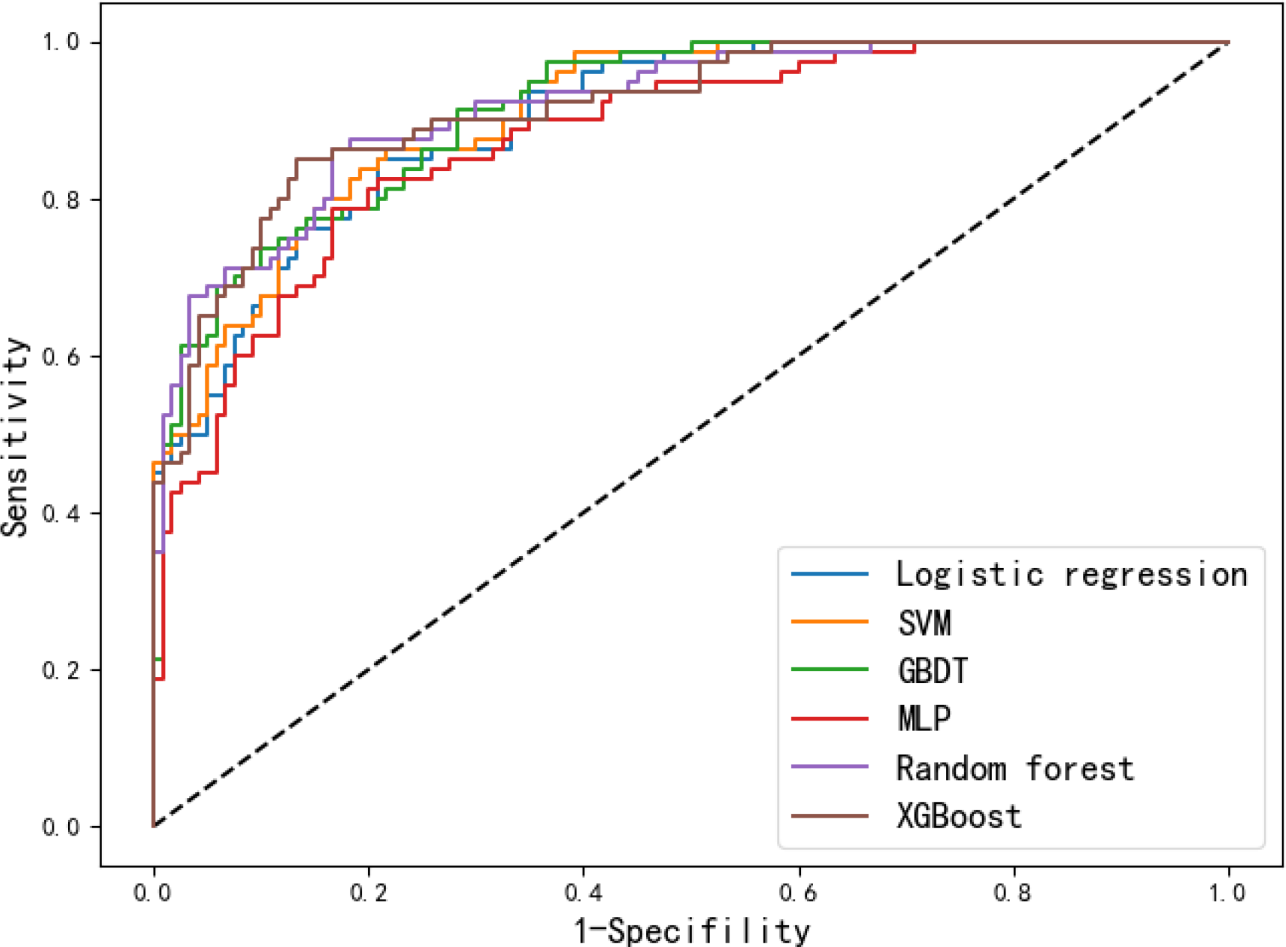
Receiver operating curves of the six models on the external validation set.

**Figure 5:**
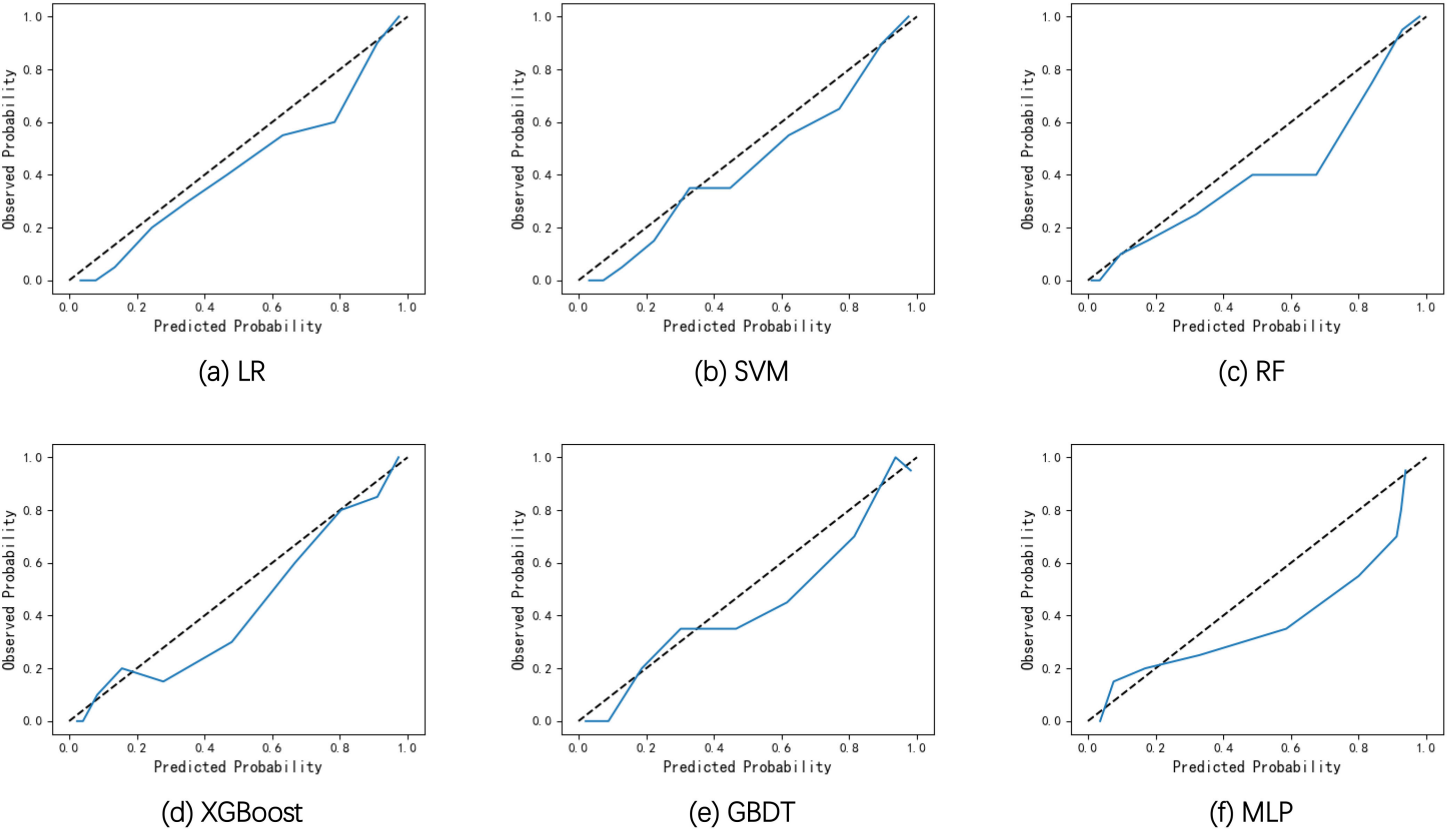
Calibration curves of the six models on the external validation set. **(A)** Calibration curves of LR. **(B)** Calibration curves of SVM. **(C)** Calibration curves of RF. **(D)** Calibration curves of XGBoost. **(E)** Calibration curves of GBDT. **(F)** Calibration curves of MLP.

The performance of the models was further evaluated on the external validation set, which were geographical independent from the training and internal validation set. The performance metrics are summarized in **Table 3**, the ROC curves and calibration curves are shown in **Figures 4** and **5** respectively. It can be seen that the performance metrics are relative stable compared to those on training and internal validation set. Although the MLP based model achieved the highest AUC value, it also produced the largest Brier score among all models. This can also be observed by the large derivation of the calibration curve from the reference curve in **Figure 5F**. The RF based model achieved the second-best AUC value and the lowest Brier score on the external validation set. Therefore, the RF based model was selected as the best model for predicting the risk of presentation delay for gastric cancer patients.

**Table 3:** Evaluation metrics of different ML models on external validation set.

Metrics	LR	SVM	RF	XGBoost	GBDT	MLP
AUC	0.901	0.910	0.917	0.915	0.914	0.927
Accuracy	0.810	0.815	0.840	0.855	0.830	0.843
Sensitivity	0.838	0.825	0.850	0.838	0.725	0.908
Specificity	0.792	0.808	0.833	0.867	0.900	0.802
PPV	0.728	0.742	0.773	0.807	0.829	0.756
NPV	0.880	0.874	0.893	0.889	0.831	0.847
F1 Score	0.779	0.781	0.810	0.822	0.773	0.765
Brier	0.130	0.123	0.118	0.118	0.120	0.158

### 3.5 Feature importance analysis

**Figure 6:**
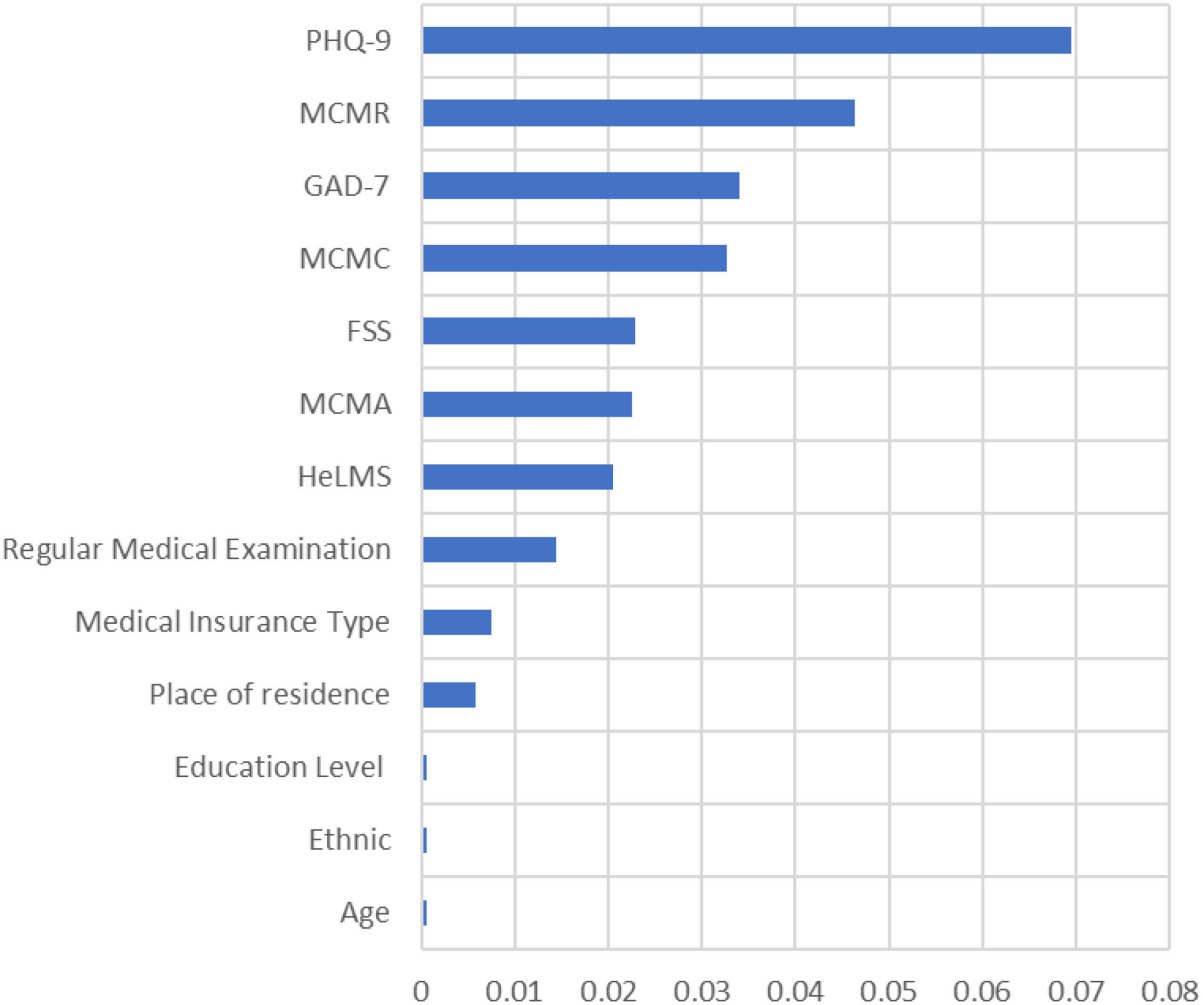
Feature importance ranking of variables.

The RF algorithm was selected as the best performed model. To better understand how different variables contribute to the prediction of presentation delay, we also ranked the features based on the feature importance values calculated based on the permutation feature importance method, as shown in **Figure 6**. According to the feature importance ranking values, the top variables were PHQ-9, GAD-7, MCMs, FSS, HeLMS. Regular health examination, medical care type and place of residence also had significant impact on the model.

## 4 Discussion

### 4.1 Current status of presentation delay in gastric cancer patients

The results of this study demonstrated that the incidence of presentation delay for gastric cancer patients was 39.3%. The result was similar to the reported 39% incidence of presentation delay for patients with multiple cancers (8), as well as the 30.0% incidence of presentation delay for the upper gastrointestinal tract (stomach and esophagus) in rural counties in the four provinces of Henan, Shandong, Jiangsu, and Anhui in China (9). This suggested we should further strengthen the publicity and education to increase residents’ knowledge of cancer and its symptoms, and promote early screening and diagnosis of cancer.

### 4.2 Risk factors of gastric cancer presentation delay

In this study, risk factors were identified according to statistical analysis of the collected data. By identify the risk factors of presentation delay of gastric cancer patients, the probability of the occurrence of presentation delay can be predicted with the ML models. Moreover, appropriate strategies can be adopted to reduce some of these risk factors.

According to the feature ranking of all risk factors, the two emotional factors the depression and anxiety score (GAD-7 and PHQ-9) ranked on top. This suggests that subjective factors have a significant impact on the occurrence of presentation delay. On one hand, the more anxious the patients are, the more they care about their own health condition. When any discomfort symptom occurs in his/her own body, the patient will be worried about that their own negligence may lead to serious consequences, and will actively seek medical treatment. On the other hand, gastric cancer patients with higher depression scores have a higher incidence of presentation delay. Emotional reactions and psychological state in cancer patients when symptoms appeared plays an important role in patients’ decision to actively seek help or admission to the hospital for consultation and treatment. Excessive worry or depression about the disease leads to fearing of diagnosis and treatment of the disease, and coping with the disease in a negative and avoiding way (35). On the contrary, patients with lower anxiety score are more likely to delay in seeking medical treatment after the appearance of symptoms. This may be due to the fact that symptoms of gastric cancer are not obvious in the early stage, only appeared as slightly gastric discomfort accompanied by acid reflux or belching. Hence the patients would not pay attention to the clinical manifestations of gastric cancer, and they don’t have enough subjective cognition of the disease. As a result, they would not be overly concerned about their own health status, and they would not seek medical treatment because of their gastric discomfort, nor would they have more anxiety because of their health problems. Therefore, we need to encourage patients to seek help and confide in family and friends, so that the uneasiness can be reduced and the depression can be alleviated.

Medical coping mode was an important predictor of the occurrence of presentation delayed in gastric cancer patients. The confrontation coping mode is an active response to the disease, the resignation coping mode shows the inability to adapt to the pain and stress brought by the disease, and the avoidance coping mode is the attitude of indifference and downplaying to the stress and pain brought by the disease. In this study, delayed gastric cancer patients had lower confrontation scores and higher resignation avoidance scores than non-delayed patients, and the difference was statistically significant. This suggested that positive facing is a protective factor against presentation delay. Positive coping mode can promote early medical treatment, shorten the delay time effectively, and improve the survival time and quality of life of patients (36). Therefore, in clinical medical care work we should help patients to regulate negative emotions and establish the correct cognition of the disease.

Family support appeared to be an important risk factor of the occurrence of presentation delay in gastric cancer patients. The higher the score of family support, the lower the incidence of presentation delay. This was in line with Zhang et al (37). who found that there was a significant correlation between patient’s delay and family support, i.e., patients who can obtain timely support from their family members and friends and receive affirmative advice on seeking medical care after detecting their symptoms can shorten their delay and increase the overall survival rate. In China, family support can give not only financial support but also emotional support to gastric cancer patients. Most gastric cancer patients will discuss with their friends and relatives before consulting a doctor, and most of the elderly people are accompanied by their children when they go to hospitals for consulting a doctor. Good family support can give the patients confidence in overcoming the disease and improve their ability to deal with inner pressure. Therefore, medical staffs should be good at finding and guiding cancer patients to actively seek effective family support in their work.

Another risk factor of presentation delay with high ranking was the health literacy of the patients. The results of this study showed that gastric cancer patients with higher health literacy scores had a lower incidence of presentation delay. Health literacy refers to an individual’s awareness and ability to maintain his or her own health. A good level of health literacy can help to improve the ability of patient to cope with diseases (38). People with lower levels of health literacy have poorer awareness and ability to maintain their own health. They often adopt negative responses when uncomfortable symptoms occur such as delay in seeking medical care, which is consistent with the results of a previous study (39). This suggests that nurses should pay more attention to and intervene in the health literacy level of people at high risk of gastric cancer, improve their awareness and ability to maintain their own health, and promote their active response to disease symptoms and timely medical treatment.

In addition to the various factors mentioned earlier, other factors that impact on the occurrence of presentation delay include regular medical examination, the medical insurance type and place of residence. Regular medical examination has significant impact on the occurrence of presentation delay. People who have regular medical examination are mostly enterprise workers or people who pay more attention to their own health. They can receive continuous health education from medical staffs during regular medical examinations. Once they find any problems they can seek medical treatment in time. Whereas those who do not have regular medical examinations have a lower awareness of their own health. They can’t catch the early warning signals, causing delay in seeking medical treatment. Among 344 cases of delayed gastric cancer patients, patients live in rural areas (253 cases, 73.5%) was significantly higher than patients live in urban area (91 cases, 26.5%). The disparity between the diagnosis and treatment of cancer between urban and rural areas had been confirmed by previous studies (40). Urban areas have richer medical resources compared with rural areas, and patients live in towns can seek medical treatment timelier as they are closer to hospitals. On the contrary, patients live in rural areas have fewer transportation options due to the remote location of rural areas. The health resources available between urban and rural areas are different as the allocation of health resources is imbalance. The difficulty of accessing medical services and the complex referral process can be barriers to seeking medical services for rural patients. Moreover, in recent years, medical and health institutions have entered cities and towns to promote cancer health education and popularize cancer prevention and treatment knowledge through many channels. But the work carried out in rural areas is insufficient. It needs to appropriately increase the allocation of health resources to rural areas, reduce the gap between regions, and ensure the accessibility of medical services in rural areas. At the same time, it is an effective countermeasure to promote timely and correct medical treatment by raising the awareness of the rural residences about cancer.

### 4.3 Comparison prediction performance of ML models

We performed a comprehensive comparison of the performance of the six ML based models. Among all six models, the RF based model had the highest AUC and Brier score and the second highest overall accuracy and F1-score, indicating it superior performance over the rest models. We noticed that the RF model has highest sensitivity but relative low specificity values among all models, which implies that the RF model has a strong ability to identify patients with high presentation delay risk and sometimes may over-estimate the risk. We considered this behavior is acceptable since our goal is to screen out high risk patients so that early intervention measures can be made to prevent serious consequences caused by presentation delay. On the contrary, a certain extent of over-estimation of the risk would not cause very bad consequences. The other two decision tree-based models, i.e. XGBoost and GBDT, achieved slightly lower AUC and overall accuracy values. This demonstrated the advantage of tree-based models for the prediction of presentation delay risk. Tree based models like RF, XGBoost or GBDT can handle various type of data flexibly. They are also with good balance between fitting ability and generalization ability by using the bagging/boosting strategy. For other models, the relatively simple linear LR model was usually used as baseline model in previous studies. In this study, the performance of LR based model was inferior to tree-based models but comparable to the SVM based model. The performance of MLP based model was inconsistent between different datasets. In summary, among the six ML based presentation delay risk prediction models for gastric cancer patients, the RF based model had the overall best performance and is suggested to be used for medical staffs for screening out patients with high presentation delay risk.

### 4.4 Limitations

Although this study was conducted in multiple centers to better verify the generalization ability of the developed machine learning models, all participating hospitals are located in western China. Therefore, the conclusions of this study may still be affected by regional differences. In addition, prospective studies are required to verify the model’s performance.

Moreover, another limitation of this study is the limited number of data samples. Training effective machine learning models with limited data is possible, as indicated by the good prediction performance on different validation datasets. Nevertheless, the number of data samples might affect the performance evaluation of different models, as complex models are more prone to overfitting with a small amount of data. For example, neural networks are generally considered as one of the most advanced machine learning methods. However, in this study the performance of MLP is found to be inferior to tree-based models. With a larger number of data samples, the performance of complex models such as MLP might be further improved. In the future, multi-center large-sample studies will be further conducted to reduce the influence of data size.

## 5 Conclusion

In this study, based on statistical analysis results 13 features were selected for predicting the risk of presentation delay in gastric cancer patients. Six machine learning based models were established and evaluated based on a dataset of 875 samples. After comparing the performance metrics of different models, the RF based model was selected as the best model. Based on the RF model, the features were ranked to demonstrated the importance for prediction presentation delay risk. It was shown that both of subjective factors such as emotional state (anxiety, depression), health literacy and objective factors such as family support, regular medical examination, place of residence had significant impact on the occurrence of presentation delay. Therefore, it is important to comprehensively assess patients’ conditions and adopt specific measures to prevent the delay of gastric cancer patients. Due to time constraints, the number of participating hospitals and the sample size of the external verification group was limited. In the future, multi-center large-sample studies or intervention studies will be further carried out to provide a basis for reducing the presentation delay of gastric cancer patients.

## Data Availability

The raw data supporting the conclusions of this article will be made available by the authors, without undue reservation.

## References

[1] Sung H Ferlay J Siegel RL Laversanne M Soerjomataram I Jemal A . Global cancer statistics 2020: GLOBOCAN estimates of incidence and mortality worldwide for 36 cancers in 185 countries. CA Cancer J Clin. (2021) 71:209–49. doi: 10.3322/caac.21660 33538338

[2] Han B Zheng R Zeng H Wang S Sun K Chen R . Cancer incidence and mortality in China, 2022. J Natl Cancer Cent. (2024) 4:47–53. doi: 10.1016/j.jncc.2024.01.006 39036382 PMC11256708

[3] Chen W Zheng R Baade PD Zhang S Zeng H Bray F . Cancer statistics in China, 2015. CA Cancer J Clin. (2016) 66:115–32. doi: 10.3322/caac.21338 26808342

[4] Subasinghe D Mahesh PKB Wijesinghe GK Sivaganesh S Samarasekera A Lokuhetty MDS . Delay in diagnosis to treatment and impact on survival of gastric adenocarcinoma in a low income setting without screening facility. Sci Rep. (2023) 13:20628. doi: 10.1038/s41598-023-47415-y 37996431 PMC10667260

[5] Pack GT Gallo JS . The culpability for delay in the treatment of cancer. Am J Cancer. (1938) 33:443–62. doi: 10.1158/ajc.1938.443

[6] Abu-Helalah MA Alshraideh HA Da'na M Al-Hanaqtah M Abuseif A Arqoob K . Delay in presentation, diagnosis and treatment for colorectal cancer patients in Jordan. J Gastrointest Cancer. (2016) 47:36–46. doi: 10.1007/s12029-015-9783-3 26615546

[7] Gyeltshen T Teh HS Loo CE Hing NYL Lim WY Subramaniam S . Factors influencing presentation delay among cancer patients: a cross-sectional study in Malaysia. BMC Public Health. (2024) 24:1260. doi: 10.1186/s12889-024-18643-2 38720253 PMC11077827

[8] Li GF Zeng LP Wang GR . Status of cancer patients delay medical treatment in China. Modern Nurs. (2008) 14:331–2. doi: 10.3760/cma.j.issn.1674-2907.2008.03.024

[9] Chen LP Zhang AH Liu HX Fan YN . Study on correlation between doctor delay and social support of cancer patients. Chin Nurs Res. (2014) 8:946–1947. doi: 10.3760/cma.j.cn115682-20230530-02150

[10] Collins GS Reitsma JB Altman DG Moons KG . Transparent reporting of a multivariable prediction model for individual prognosis or diagnosis (TRIPOD): the TRIPOD statement. BMJ. (2015) 350:g7594. doi: 10.1136/bmj.g7594 25569120

[11] Hart GR Yan V Huang GS Liang Y Nartowt BJ Muhammad W . Population-based screening for endometrial cancer: human vs. Machine intelligence. Front Artif Intell. (2020) 3:539879. doi: 10.3389/frai.2020.539879 33733200 PMC7861326

[12] Stark GF Hart GR Nartowt BJ Deng J . Predicting breast cancer risk using personal health data and machine learning models. PLoS One. (2019) 14:e0226765. doi: 10.1371/journal.pone.0226765 31881042 PMC6934281

[13] O'Connor S Vercell A Wong D Yorke J Fallatah FA Cave L . The application and use of artificial intelligence in cancer nursing: A systematic review. Eur J Oncol Nurs. (2024) 68:102510. doi: 10.1016/j.ejon.2024.102510 38310664

[14] Du J Yang J Yang Q Zhang X Yuan L Fu B . Comparison of machine learning models to predict the risk of breast cancer-related lymphedema among breast cancer survivors: a cross-sectional study in China. Front Oncol. (2024) 14:1334082. doi: 10.3389/fonc.2024.1334082 38410115 PMC10895296

[15] Zuo D Yang L Jin Y Qi H Liu Y Ren L . Machine learning-based models for the prediction of breast cancer recurrence risk. BMC Med Inform Decis Mak. (2023) 23:276. doi: 10.1186/s12911-023-02377-z 38031071 PMC10688055

[16] Mikulin T Hardcastle JD . Gastric cancer-delay in diagnosis and its causes. Eur J Cancer Clin Oncol. (1987) 23:1683–90. doi: 10.1016/0277-5379(87)90450-0 3428334

[17] Macdonald S Macleod U Campbell NC Weller D Mitchell E . Systematic review of factors influencing patient and practitioner delay in diagnosis of upper gastrointestinal cancer. Br J Cancer. (2006) 94:1272–80. doi: 10.1038/sj.bjc.6603089 PMC2361411 16622459

[18] Mazidimoradi A Momenimovahed Z Salehiniya H . Barriers and facilitators associated with delays in the diagnosis and treatment of gastric cancer: A systematic review. J Gastrointest Cancer. (2022) 53:782–96. doi: 10.1007/s12029-021-00673-3 34499307

[19] Dehdar S Salimifard K Mohammadi R Marzban M Saadatmand S Fararouei M . Applications of different machine learning approaches in prediction of breast cancer diagnosis delay. Front Oncol. (2023) 13:1103369. doi: 10.3389/fonc.2023.1103369 36874113 PMC9978377

[20] Frosch ZAK Hasler J Handorf E DuBois T Bleicher RJ Edelman MJ . Development of a multilevel model to identify patients at risk for delay in starting cancer treatment. JAMA Netw Open. (2023) 6:e2328712. doi: 10.1001/jamanetworkopen.2023.28712 37578796 PMC10425824

[21] Wang GR Jiang XL Wang LP . Research on status quo and intervention on delay to see doctors of breast cancer patients. Chin Nurs Researsh. (2007) 21:1979–81. doi: 10.3969/j.issn.1009-6493.2007.22.003

[22] Procidano ME Heller K . Measures of perceived social support from friends and from family: three validation studies. Am J Community Psychol. (1983) 11:1–24. doi: 10.1007/BF00898416 6837532

[23] Jordan JE Buchbinder R Briggs AM Elsworth GR Busija L Batterham R . The health literacy management scale (HeLMS): a measure of an individual's capacity to seek, understand and use health information within the healthcare setting. Patient Educ Couns. (2013) 91:228–35. doi: 10.1016/j.pec.2013.01.013 23419326

[24] Sun HL Peng H Fu H . The reliabililty and consistency of health literacy scale for chronic patients. Fudan Univ J Med Sci. (2012) 39:268–72. doi: 10.3969/j.issn.1672-8467.2012.03.009

[25] Rodrigue JR Jackson SI Perri MG . Medical coping modes questionnaire: Factor structure for adult transplant candidates. Int J Behav Med. (2000) 7:89–110. doi: 10.1207/S15327558IJBM0702_1

[26] Shen XH Jiang QJ . Report on application of Chinese version of MCMQ in 701 patients. Chin J Behav Med Sci. (2000) 9:18–20. doi: 10.3760/cma.j.issn.1674-6554

[27] Spitzer RL Kroenke K Williams JB Löwe B . A brief measure for assessing generalized anxiety disorder: the GAD-7. Arch Intern Med. (2006) 166:1092–7. doi: 10.1001/archinte.166.10.1092 16717171

[28] Kroenke K Spitzer RL Williams JB . The PHQ-9: validity of a brief depression severity measure. J Gen Intern Med. (2001) 16:606–13. doi: 10.1046/j.1525-1497.2001.016009606.x PMC1495268 11556941

[29] Lorena AC Jacintho LFO Siqueira MF de Giovanni R Lohmann LG de Carvalho ACPLF . Comparing machine learning classifiers in potential distribution modelling. Expert Syst Appl. (2011) 38:5268–75. doi: 10.1016/j.eswa.2010.10.031

[30] Cortes C Vapnik V . Support-vector networks. Mach Learn. (1995) 20:273–97. doi: 10.1023/A:1022627411411

[31] Breiman L . Random forests. Mach Learn. (2001) 45:5–32. doi: 10.1023/A:1010933404324

[32] Chen T Guestrin C . XGBoost: a scalable tree boosting system. In: Proceedings of the 22nd ACM SIGKDD International Conference on Knowledge Discovery and Data Mining. San Francisco, California, USA: Association for Computing Machinery (2016). doi: 10.1145/2939672.2939785

[33] Friedman JH . Greedy function approximation: a gradient boosting machine. Ann Stat. (2001) 29:1189–232. doi: 10.1214/aos/1013203451

[34] Safara AA Salihb DM Murshid AM . Pattern recognition using the multi-layer perceptron (MLP) for medical disease: A survey. Int J Nonlinear Anal Appl. (2023) 14:1989–98. doi: 10.22075/IJNAA.2022.7114

[35] He QL Li DD Wang XH Bi YX . Investigation and analysis of influencing factors of delayed medical treatment in patients with oral cancer and intervention measures. J Clin Nurs. (2020) 19:8–11. doi: 10.3969/j.issn.1671-8933.2020.06.003

[36] Liu Z Zhang L Cao Y Xia W Zhang L . The relationship between coping styles and benefit finding of Chinese cancer patients: The mediating role of distress. Eur J Oncol Nurs. (2018) 34:15–20. doi: 10.1016/j.ejon.2018.03.001 29784133

[37] Zhang H Wang G Zhang J Lu Y Jiang X . Patient delay and associated factors among Chinese women with breast cancer: A cross-sectional study. Medicine. (2019) 98:e17454. doi: 10.1097/MD.0000000000017454 31577773 PMC6783180

[38] Moore C Hassett D Dunne S . Health literacy in cancer caregivers: a systematic review. J Cancer Surviv. (2021) 15:825–36. doi: 10.1007/s11764-020-00975-8 33409857

[39] Fairfield KM Black AW Lucas FL Murray K Ziller E Korsen N . Association between rurality and lung cancer treatment characteristics and timeliness. J Rural Health. (2019) 35:560–5. doi: 10.1111/jrh.12355 30779871

[40] Bhatia S Landier W Paskett ED Peters KB Merrill JK Phillips J . Rural-urban disparities in cancer outcomes: opportunities for future research. J Natl Cancer Inst. (2022) 114:940–52. doi: 10.1093/jnci/djac030 PMC9275775 35148389

